# Assessing time to pulmonary function benefit following antibiotic treatment of acute cystic fibrosis exacerbations

**DOI:** 10.1186/1465-9921-11-137

**Published:** 2010-10-06

**Authors:** Donald R VanDevanter, Mary A O'Riordan, Jeffrey L Blumer, Michael W Konstan

**Affiliations:** 1 Case Western Reserve University School of Medicine, Cleveland, OH, USA; 2 Rainbow Babies and Children's Hospital, Cleveland, OH, USA

## Abstract

**Background:**

Cystic Fibrosis (CF) is a life-shortening genetic disease in which ~80% of deaths result from loss of lung function linked to inflammation due to chronic bacterial infection (principally *Pseudomonas aeruginosa*). Pulmonary exacerbations (intermittent episodes during which symptoms of lung infection increase and lung function decreases) can cause substantial resource utilization, morbidity, and irreversible loss of lung function. Intravenous antibiotic treatment to reduce exacerbation symptoms is standard management practice. However, no prospective studies have identified an optimal antibiotic treatment duration and this lack of objective data has been identified as an area of concern and interest.

**Methods:**

We have retrospectively analyzed pulmonary function response data (as forced expiratory volume in one second; FEV_1_) from a previous blinded controlled CF exacerbation management study of intravenous ceftazidime/tobramycin and meropenem/tobramycin in which spirometry was conducted daily to assess the time course of pulmonary function response.

**Results:**

Ninety-five patients in the study received antibiotics for at least 4 days and were included in our analyses. Patients received antibiotics for an average of 12.6 days (median = 13, SD = 3.2 days), with a range of 4 to 27 days. No significant differences were observed in mean or median treatment durations as functions of either treatment group or baseline lung disease stage. Average time from initiation of antibiotic treatment to highest observed FEV_1 _was 8.7 days (median = 10, SD = 4.0 days), with a range of zero to 19 days. Patients were treated an average of 3.9 days beyond the day of peak FEV_1 _(median = 3, SD = 3.8 days), with 89 patients (93.7%) experiencing their peak FEV_1 _improvement within 13 days. There were no differences in mean or median times to peak FEV_1 _as a function of treatment group, although the magnitude of FEV_1 _improvement differed between groups.

**Conclusions:**

Our results suggest that antibiotic response to exacerbation as assessed by pulmonary function is essentially complete within 2 weeks of treatment initiation and relatively independent of the magnitude of pulmonary function response observed.

## Introduction

Cystic Fibrosis (CF) is a life-shortening genetic disease in which ~80% of deaths result from loss of lung function linked to inflammation caused by chronic bacterial lung infection (principally *Pseudomonas aeruginosa*) [1,2]. Pulmonary exacerbations (intermittent episodes during which symptoms of lung infection increase and lung function decreases) can cause substantial resource utilization [3], morbidity [4], and irreversible loss of lung function [5].

A number of different signs, symptoms, and test results can contribute to the diagnosis of CF pulmonary exacerbation [2,4], with precipitous loss of forced expiratory volume in 1 second (FEV_1_) most strongly associated with exacerbation diagnosis in patients old enough to reliably perform spirometry [6]. Because loss of lung function is the underlying cause of a majority of CF deaths [1], management of CF exacerbations includes an understandable emphasis on recovery of lung function [7]. Unfortunately, many patients fail to fully recover lost lung function despite this emphasis. One study has shown that 43% of patients treated for pulmonary exacerbation failed to completely reach their pre-exacerbation pulmonary function, and 24% failed to achieve even 95% of their pre-exacerbation function [5].

Administration of systemic antibiotics is an essential component of CF exacerbation management, particularly in patients chronically infected with *Pseudomonas aeruginosa *[7,8]. Three blinded placebo-controlled clinical studies failed to demonstrate a significant antipseudomonal antibiotic effect on increased respiratory signs and symptoms associated with exacerbation [9-11] and only one of these studies could attribute a significant improvement in FEV_1 _% predicted to antibiotic treatment [11]. Regardless, treatment guidelines suggest that antibiotics be administered for a CF exacerbation "for a minimum of 10 days but often will be extended depending on the time course until clinical improvement is seen and the improvement or lack thereof, of pulmonary function tests" [7]. This guidance, which is based more on experience and consensus than evidence, does not address the question of when antibiotic regimens should be adjusted or abandoned entirely in the absence of complete recovery after extended treatment. A recent Cochrane review noted this problem and suggested that a randomized controlled trial comparing different antibiotic treatment durations could have important clinical and financial implications [12]. Similarly, a recent CF Foundation consensus document concluded that "there is insufficient evidence to recommend an optimal duration of antibiotic treatment of an acute exacerbation," noting that duration of therapy is "an important question that should be studied further" [8].

We have retrospectively analyzed data from a previous study of antibiotic treatment of CF exacerbation in which spirometry was conducted daily [13]. In this multicenter study, treatment duration was left to the individual investigator's discretion based upon their current standards of exacerbation management. Our purpose was to characterize FEV_1 _response to antibiotic therapy as a function of time to determine if FEV_1 _response continued or was attenuated with extended treatment.

## Methods

Data were obtained from a previous blinded, randomized study comparing treatment with intravenous ceftazidime plus tobramycin (ceftaz/tobra) to treatment with meropenem plus tobramycin (mero/tobra) for acute pulmonary exacerbation [13]. This previous study was conducted in accordance with the recommendations found in the Helsinki Declaration of 1975. The study protocol and informed consent were approved by the Institutional Review Board for Human Studies at each site. Informed consent was obtained from each subject. To be included in the current analyses, patients had to have been enrolled in the blinded, randomized portion of the previous study, have had spirometry performed at treatment initiation (Day 0) and have received four or more consecutive days of treatment with ceftaz/tobra or mero/tobra from initiation. Baseline lung disease stage ("early," FEV_1 _≥ 70% predicted; "intermediate," FEV_1 _between 40% and 69% predicted; "advanced," FEV_1 _< 40% predicted), antibiotic treatment assignments, and all available daily spirometric measures beginning at Day 0 were collected for each patient. Relative change in FEV_1 _was calculated by subtracting a patient's Day 0 FEV_1 _value from the FEV_1 _value on any subsequent treatment day, and then dividing by the Day 0 value. Relative change values were expressed as percentages. Peak change in FEV_1 _was defined as the largest relative increase in FEV_1 _observed after initiation of treatment for each subject. Differences in median treatment durations and elapsed times from treatment initiation to observation of peak FEV_1 _were studied as functions of antibiotic treatment received and baseline lung disease stage by Wilcoxon rank sum tests. Differences in absolute and relative change in FEV_1 _from baseline between groups were analyzed by t tests.

## Results

**Figure 1:**
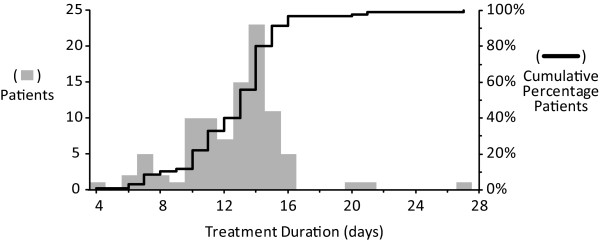
**Distribution of antibiotic treatment durations**. The gray bars show the distribution of antibiotic treatment duration in days for the 95 study patients (left vertical axis). The black line represents the cumulative percentage of patients having completed treatment by a given duration (right vertical axis).

Ninety-five patients (50 treated with ceftaz/tobra and 45 treated with mero/tobra) met inclusion criteria and were included in this retrospective analysis. At initiation of treatment, 11 patients (11.6%) had an FEV_1 _of ≥70% predicted, 46 (48.4%) had an FEV_1 _of between 40% and 70% predicted, and 38 (40.0%) had an FEV_1 _of < 40% predicted. Baseline FEV_1 _values (in liters) for subgroups are provided in Table 1. Patients were treated with antibiotics for an average of 12.6 days (median = 13, SD = 3.2 days), with a range of 4 to 27 days (Table 1). Seventy-six patients (80%) had completed antibiotic treatment by 14 days, and 96.8% had completed treatment by 16 days (Figure 1). No significant differences were observed in mean or median antibiotic treatment durations as functions of either antibiotic treatment received or baseline lung disease stage, although there was a trend towards longer median treatment durations with mean greater impairment of baseline FEV_1 _(Table 1).

**Table 1:** Treatment Duration and Time to Peak FEV_1 _Measure

	Mean, days ± SD	Median, days	Range, days (min - max)	Interquartile Range, days
**Antibiotic Treatment Duration**				
All patients (n = 95)	12.6 ± 3.2	13	23 (4 - 27)	3
ceftaz/tobra treated (n = 50)	12.5 ± 2.8	12.5	12 (4 - 16)	3.75
mero/tobra treated (n = 45)	12.8 ± 3.6	13	21 (6 - 27)	3
FEV_1 _≥ 70% predicted (n = 11)	11.9 ± 2.4	12	8 (7 - 15)	3
FEV_1 _40% - 69% predicted (n = 46)	12.3 ± 2.8	13	15 (6 - 21)	3
FEV_1 _< 40% predicted (n = 38)	13.2 ± 3.8	14	23 (4 - 27)	3
**Time to Peak FEV_1 _Measure**				
All patients	8.7 ± 4.0	10	19 (0 - 19)	6.5
ceftaz/tobra treated	8.9 ± 4.0	10	15 (1 - 16)	6.75
mero/tobra treated	8.5 ± 4.3	9	19 (0 - 19)	5
FEV_1 _≥ 70% predicted	7.9 ± 3.3	7	12 (4 - 15)	5
FEV_1 _40% - 69% predicted	8.0 ± 4.2	9.5	15 (0 - 15)	6
FEV_1 _< 40% predicted	9.9 ± 3.6	10	16 (3 - 19)	4.75

**Figure 2:**
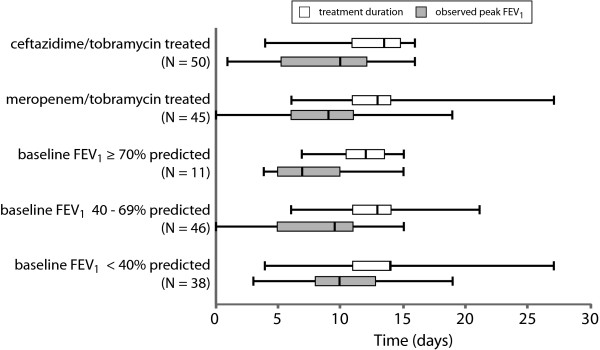
**Treatment duration and time to peak response by subgroups**. Box and whisker plots of antibiotic treatment duration (white boxes) and time to peak observed FEV_1 _measure (gray boxes) among patients stratified by antibiotic treatment (ceftaz/tobra and mero/tobra) and by lung disease stage (FEV_1 _≥ 70% predicted, 40- 69% predicted, and <40% predicted).

**Figure 3:**
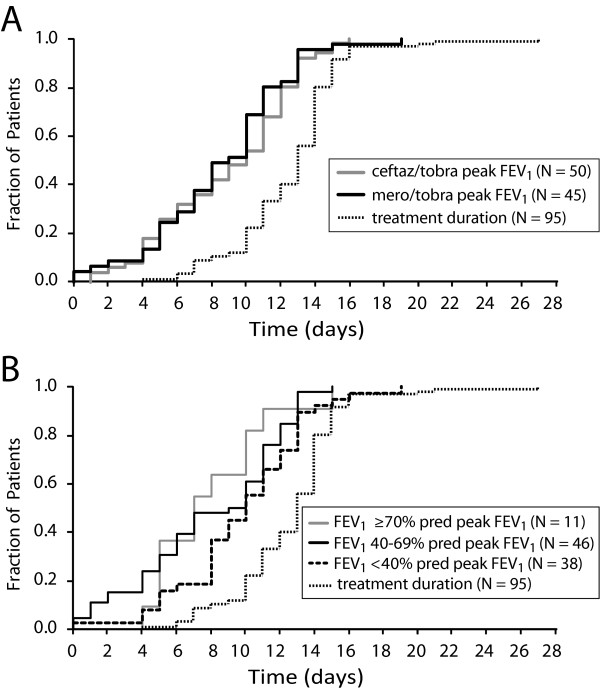
**Time from antibiotic treatment initiation to peak FEV_1 _measure for subgroups**. Panel A, Time to peak FEV_1 _measure stratified by antibiotic treatment assignment. Gray line, ceftaz/tobra; black line, mero/tobra; dotted line, population treatment duration. Panel B. Time to peak FEV_1 _measure stratified by baseline lung function. Gray line, patients with baseline FEV_1 _≥ 70% predicted, black line, patients with baseline FEV_1 _between 40% and 69% predicted; dashed line, patients with baseline FEV_1 _< 40% predicted; dotted line, population treatment duration.

The average time from initiation of antibiotic treatment to the highest observed FEV_1 _for all patients was 8.7 days (median = 10, SD = 4.0 days), with a range of zero to 19 days (Table 1). Two patients (2.1%) did not experience an increase in FEV_1 _over their baseline values, despite being treated with antibiotics for 8 and 15 days. Twelve patients (12.6%) experienced their greatest relative improvement in FEV_1 _on their final day of antibiotic treatment, which occurred on average 11.5 days after initiation (median = 11, SD = 3.1 days). Patients were treated with antibiotics for an average of 3.9 days beyond the day that their peak FEV_1 _was observed (median = 3, SD = 3.8 days). The patient treated for the longest duration (27 days) was also the patient treated for the longest period after recording his or her peak FEV_1 _(at day 5). In all, 70 patients (73.7%) had already experienced their peak improvement in FEV_1 _by 11 days of antibiotic treatment, and 89 patients (93.7%) had experienced their peak improvement in FEV_1 _by 13 days of treatment. There was no significant difference observed in median time to peak FEV_1 _response observed between patients treated with ceftaz/tobra and those treated with mero/tobra (10 versus 9 days, P = 0.52)(Figures 2 and 3A). In contrast, mean time to peak FEV_1 _response was impacted by baseline lung disease stage, with patients entering the study with an FEV_1 _< 40% predicted requiring a significantly longer median treatment duration to achieve their peak FEV_1 _response compared with those patients with a baseline FEV_1 _between 40% and 69% predicted (8.0 versus 9.9 days, P = 0.041) (Figures 2 and 3B).

For the entire population, patients experienced an average peak increase in FEV_1 _of 0.55 liters, from a baseline average of 1.40 liters to a peak average of 1.95 liters (Table 2). In relative terms, patients experienced an average 47.2% increase over their baseline FEV_1 _at peak. Patients receiving mero/tobra experienced a greater average increase in FEV_1 _from baseline to their peak measure than patients receiving ceftaz/tobra (0.65 liters versus 0.46 liters; p = 0.033). However, when individual peak FEV_1 _increases were normalized for baseline FEV_1 _values, the 55.9% relative increase experienced by mero/tobra patients was not significantly different (p = 0.056) than the 39.3% relative increase experienced by ceftaz/tobra patients. Average peak increases in FEV_1 _were not significantly different for patients with different baseline lung disease stages, ranging from 0.51 liters to 0.70 liters (Table 2).

**Table 2:** FEV_1 _Measures

Subject Group	Mean, liters ± SD	Median, liters	Range, liters (min - max)
**Baseline FEV_1_**			
All patients (n = 95)	1.40 ± 0.71	1.19	3.09 (0.44 - 3.53)
ceftaz/tobra treated (n = 50)	1.37 ± 0.71	1.13	3.09 (0.44 - 3.53)
mero/tobra treated (n = 45)	1.44 ± 0.71	1.37	2.99 (0.48 - 3.47)
FEV_1 _≥ 70% predicted (n = 11)	2.46 ± 0.87	2.72	2.38 (1.15 - 3.53)
FEV_1 _40% - 69% predicted (n = 46)	1.54 ± 0.56	1.47	2.52 (0.56 - 3.08)
FEV_1 _< 40% predicted (n = 38)	0.93 ± 0.30	0.90	1.43 (0.44 - 1.87)
**Observed Peak FEV_1_**			
All patients	1.95 ± 0.87	1.72	3.62 (0.67 - 4.29)
ceftaz/tobra treated	1.83 ± 0.85	1.55	3.29 (0.67 - 3.96)
mero/tobra treated	2.09 ± 0.88	1.78	2.99 (0.48 - 4.29)
FEV_1 _≥ 70% predicted	3.17 ± 1.01	3.71	2.99 (1.30 - 4.29)
FEV_1 _40% - 69% predicted	2.05 ± 0.71	2.05	2.76 (0.84 - 3.60)
FEV_1 _< 40% predicted	1.48 ± 0.59	1.39	2.44 (0.67 - 3.11)
**FEV_1 _Change, Baseline to Peak**			
All patients	0.55 ± 0.43	0.46	2.19 (0.0 - 2.19)
ceftaz/tobra treated	0.46 ± 0.36	0.39	1.50 (0.05 - 1.55)
mero/tobra treated	0.65 ± 0.49	0.55	2.19 (0.0 - 2.19)
FEV_1 _≥ 70% predicted	0.70 ± 0.46	0.43	1.40 (0.15 - 1.55)
FEV_1 _40% - 69% predicted	0.51 ± 0.41	0.46	2.19 (0.0 - 2.19)
FEV_1 _< 40% predicted	0.56 ± 0.45	0.43	1.93 (0.01 - 1.94)

## Discussion

There may be no more challenging aspect of CF exacerbation management than determining an "appropriate" or "optimal" use of systemic antibiotics [8]. In addition to the question of which organisms within complex multispecies lung infections should be targeted (and by extension, which specific antibiotics should be chosen), the question of how long antibiotics should be administered has yet to be meaningfully explored [8,12]. In the past, CF treatment guidelines have suggested that treatment duration should be determined empirically by "the improvement, or lack thereof" of pulmonary function [7]. This suggestion is logical, in that loss of FEV_1 _is a strong predictor of exacerbation diagnosis [6], and antibiotic therapy has been shown to significantly improve lung function in CF patients experiencing an exacerbation [11]. Unfortunately, many patients treated for a CF exacerbation do not fully recover lung function lost immediately prior to intervention [5], creating a dilemma for the treating clinician: if a patient has not fully recovered lost lung function following weeks of antibiotic treatment, should treatment be extended? At what point does the clinician accept that the patient will not fully recover lung function on the current regimen, and that continued antibiotic treatment may be futile and possibly deleterious?

The reasons that some patients fail to completely recover lost lung function following exacerbation [5] remain unknown. It may be that antibiotic choices and/or treatment durations made for some patients have been suboptimal, or simply that irreversible lung damage has occurred in some patients, or both. It can be argued, however, that an "optimal" duration for *any *antibiotic therapy would be one that includes an observed peak increase in FEV_1_, regardless of the magnitude of the increase observed. In practice, it is impossible to recognize that moment in time when a patient is experiencing his or her peak FEV_1 _improvement, as this is an inherently retrospective analysis. It is possible, however, to retrospectively review a series of treatment courses and ask whether the timing of peak responses adhere to a consistent pattern that could be useful in predicting future response time courses. Recently, a retrospective analysis of 1,535 patients treated for exacerbation while participating in the US CF Twin and Sibling Study between 2000-2007 showed that, on average, lung function recovery reached a plateau after 8 and 10 days of treatment [14] a result similar to that observed in a small prospective study by Regelmann et al. [11] two decades earlier. Our results compliment and expand on these earlier findings, in that we have been able to analyze daily PFT measures for individual patients to confirm that attainment of peak FEV_1 _occurs fairly consistently within 2 weeks of initiation of antibiotic treatment.

There are several limitations to the current analysis, not the least of which is that patients were not consistently treated for an extended duration (e.g., 3 or more weeks) in order to derive more accurate time to peak FEV_1 _response curves. It is possible that if all patients (and particularly those treated for the shortest durations) had been treated longer, they may have experienced higher peak FEV_1 _values later in treatment. For instance, the mean peak improvement in FEV_1 _for the 10 patients treated between 4 and 8 days was only 0.46 liters (median = 0.26, SD = 0.64 liters) (Table 3), lower than the population average of 0.55 liters (Table 2). However, only 2 of these patients experienced their peak FEV_1 _on their final day of treatment, and although the median treatment duration within this subgroup was 7 days, the median time to peak FEV_1 _response among these patients was only 4.5 days. Comparison of outcomes for the 43 patients treated between 9 and 13 days with the 42 patients treated for 14 days or longer suggests that little advantage was obtained by extended treatment, with 85.7% of patients treated for greater than 14 days experiencing their peak FEV_1 _before 14 days treatment (Table 3). Average peak FEV_1 _improvement trended lower in patients treated for more than 14 days compared to those treated for 9 to 13 days (0.50 versus 0.62 liters; P = 0.17). Average peak FEV_1 _improvement for the 8 patients treated for at least 16 days was 0.40 liters (median = 0.36, SD = 0.20 liters), with half experiencing their peak FEV_1 _by day 12. Although investigators may have extended the treatment of these patients on the basis of a poor initial FEV_1 _response, extending treatment did not result with these patients having a higher overall FEV_1 _response than others in the study.

**Table 3:** Relationships between Treatment Duration and Observed Peak FEV_1_

	Treatment Duration Window		
	**4-8 Days**	**9-13 Days**	**14+ Days**
Patients, n	10	43	42
Median Treatment Duration, days	7	12	14
Median Peak FEV_1 _Observation, days	4.5	8	11.5
Mean Peak FEV_1 _Increase, liters ± SD	0.46 ± 0.64	0.62 ± 0.43	0.50 ± 0.38
Patients Reaching Peak FEV_1 _*before *Duration Window, n (%)	3 (30.0%)	22 (51.2%)	36 (85.7%)

A caveat of this retrospective study is that patients had to have had at least one ceftazidime and meropenem susceptible strain of *P. aeruginosa *isolated from their respiratory secretions at baseline in order to be eligible for randomization [13]. Although it has been suggested previously that response to antipseudomonal antibiotics (and particularly to ceftazidime plus tobramycin) is independent of *in vitro *susceptibility test results in CF [15], it may be that time to peak FEV_1 _response may be impacted by the absence of antibiotic susceptible strains in patients experiencing an exacerbation.

**Figure 4:**
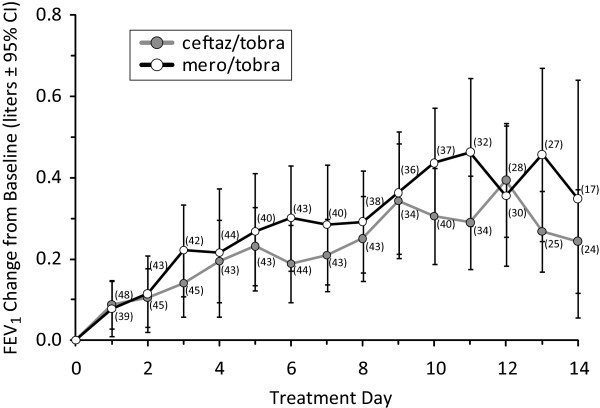
**Average daily change in FEV_1 _from baseline by treatment group**. Gray circles, patients treated with ceftaz/tobra. Open circles, patients treated with mero/tobra. Sample sizes for measures are in parentheses.

It is not our intention to discourage treating physicians from diligently pursuing other treatable causes of failure to improve FEV_1 _to pre-exacerbation average or recent best FEV_1 _in patients who have experienced pulmonary exacerbation. However, these data suggest that continued application of a given antibiotic intervention beyond approximately two weeks is unlikely to result in additional patient benefit with respect to FEV_1_. Results from this retrospective analysis suggest hypotheses that might be tested in prospective CF exacerbation clinical trials. First, median times to peak FEV_1 _response for the two antibiotic treatments were not significantly different (Figures 2 and 3A) despite a suggestion of a difference in the magnitude of peak FEV_1 _response to each treatment (Table 2 and Figure 4). This result implies that antibiotic treatment duration and FEV_1 _response magnitude may be relatively independent, and that the magnitude of an antibiotic response is unlikely to be improved by extending treatment beyond 14 days. The profiles of mean daily changes in FEV_1 _from baseline through day 14 by treatment group are consistent with this hypothesis, in that the relative amplitudes of the profiles differ but their shapes are similar (Figure 4). A blinded study in which subjects are randomized to receive either 14 days of treatment or the current practice of extended treatment at clinician discretion [7] could address the question of whether additional benefit (or harm) is associated with extended treatment. Second, 89 of 95 patients (93.7%) experienced their highest FEV_1 _on or before day 13 of treatment, and only 12 (12.6%) patients experienced their peak FEV_1 _on their last day of treatment, including 7 who were treated less than 12 days. These results suggest that limiting treatment protocols to 14 days duration for the purposes of comparing responses to different antibiotic treatments would be both ethical and likely to detect true differences between treatments. Third, there was a noticeable trend with respect to the impact of baseline lung function on time to peak FEV_1 _response, with patients having a baseline FEV_1 _≥ 70% predicted having a significantly shorter median time to peak response (7 days) when compared to patients with baseline FEV_1 _< 40% predicted (10 days, P = 0.041) (Table 1; Figures 2 and 3B). Interestingly, the *magnitude *of improvement in FEV_1 _(in liters) was fairly consistent across lung disease stages (Table 2). These results suggest that baseline lung disease stage should be accounted for in the design and analysis of prospective exacerbation studies. Finally, our analyses have necessarily been limited to the question of when peak FEV_1 _was observed during antibiotic treatment, but the implication that FEV_1 _increase following exacerbation is limited to that period when antibiotics are administered may not be justified. Unfortunately, subject spirometry after cessation of antibiotics was not available for this retrospective analysis, but a prospective trial of antibiotic treatment duration should consider the possibility that additional recovery of FEV_1 _may occur after antibiotic treatment is halted.

## Abbreviations Used

Ceftaz: ceftazidime; CF: cystic fibrosis; FEV_1_: forced expiratory volume in 1 second; mero: meropenem; PFT: pulmonary function test; tobra: tobramycin

## Competing interests

The authors declare that they have no competing interests.

## Authors' contributions

JB was the principal investigator on the original randomized controlled trial. DV and MK conceived of the retrospective study and DV conducted the analyses and constructed the figures. MO provided data management support and reviewed/contributed to the statistical analysis plan. DV drafted the manuscript and all authors contributed to the revision and finalization of the manuscript. All authors approved the final manuscript.
